# Town Mouse or Country Mouse: Identifying a Town Dislocation Effect in Chinese Urbanization

**DOI:** 10.1371/journal.pone.0125821

**Published:** 2015-05-14

**Authors:** Fei Wang, Shu Li, Xin-Wen Bai, Xiao-Peng Ren, Li-Lin Rao, Jin-Zhen Li, Huan Liu, Hong-Zhi Liu, Bin Wu, Rui Zheng

**Affiliations:** 1 Key Laboratory of Behavioral Science, Institute of Psychology, Chinese Academy of Sciences, Beijing, China; 2 School of Journalism and Communication, Xiamen University, Xiamen, China; 3 Management School, Jinan University, Guangzhou, China; 4 Center for Mental Health, Nanchang University, Nanchang, China; TNO, NETHERLANDS

## Abstract

Understanding urbanization and evaluating its impact are vital for formulating global sustainable development. The results obtained from evaluating the impact of urbanization, however, depend on the kind of measurement used. With the goal of increasing our understanding of the impact of urbanization, we developed direct and indirect subjective indicators to measure how people assess their living situation. The survey revealed that the projected endorsements and perceived social ambiance of people toward living in different types of settlements did not improve along with the urbanization level in China. The assessment scores from the city dwellers were not significantly different from those from the country areas and, more surprisingly, both were significantly higher than the assessment scores of the town dwellers, which we had expected to fall between the assessment scores of the country and city dwellers. Instead their scores were the lowest. We dubbed this V-shaped relationship the “town dislocation effect.” When searching for a potential explanation for this effect, we found additional town dislocation effects in social support, loss aversion, and receptivity toward genetically modified food. Further analysis showed that only social support mediated the relationship between the three tiers of settlements (cities, country areas, and towns) and the subjective indicator. The projected endorsements yielded significant subjective assessments that could enhance our understanding of Chinese urbanization. Towns posed specific problems that require special attention.

## Introduction

*Better beans and bacon in peace than cakes and ale in fear*.

— Aesop

*The Town Mouse and the Country Mouse* is an Aesop fable which told the story of a town mouse and a country mouse who were deciding where to live. The two mice had different assessment criteria: The town mouse used objective or hard assessment criteria and preferred the city with plenty of cakes and ale, whereas the country mouse used subjective or soft criteria and preferred his safe bare plow lands without any fear. This fable could be taken to imply that people’s assessment of their settlement locations will vary between different types of indicators. Adopting objective or hard indicators could lead to town-preferred choices, whereas adopting subjective or soft indicators could lead to country-preferred choices. The mice’s final decision of whether to live or not to live in a town, which was revealed by voting with their feet, is more likely to be determined by subjective criteria rather than objective ones.

Country areas, towns, and cities are the three main tiers of settlement in the urbanization process [1]. Half of the entire world population now resides in cities, and three million people are migrating to the cities of the developing world every week [2]. Approximately 70% of the entire world population is expected to reside in cities by the middle of the century [3]. The ultimate purpose of urbanization is to improve the quality of people’s lives [4]. Therefore, it is critical to identify the effects of urbanization on people’s quality of life. Previous studies on the effects of urbanization primarily focused on objective or hard indicators, such as productivity, infrastructure development [3], economic growth [5], climate change [6–9], environment and ecosystems [10–13], education, health, birth rate, and life span [14]. Only a few research studies have used subjective indicators [14] such as self-reported social well-being, psychosocial well-being [15], and rated quality of life [16]. Objective indicators reveal little about residents’ inner perspectives about whether urbanization really improves their quality of life [17]. Therefore, the current study focuses on the effects of urbanization on subjective indicators such as safety, as referred to by Aesop in the abovementioned fable.

This story by Aesop suggested that the best place to live in is not the place filled with cakes and ale but in the place where inhabitants can live happily. The desirability of a local settlement is typically determined by the perceived social ambiance of inhabitants toward the location of their settlement [18–20]. Therefore, we included social ambiance as a subjective indicator in the current study. The ratings of social ambiance were primarily based on the concept of harmonious cities proposed by UN-HABITAT [2], which suggested certain aspects which are important to sustainable urban development. These were the good governance that the settlement provides, the tolerance that the settlement promotes, and the fairness and social justice that the settlement aims to achieve [2]. In addition, because “safety” and “social equality” have both been investigated in studies of what constitutes a good society [21–23], we added these two aspects to the above four aspects from the UN-HABITAT’s concept of harmonious cities. Thus, these six aspects were assessed to measure the rated social ambiance. Taken together, these presumably can define the character and atmosphere of current Chinese society.

We likewise used a type of projective method to measure whether inhabitants endorse their own place of settlement. Projective methods can potentially identify the innermost thoughts and feelings of an individual or the essence of his or her individuality [24]. They involve the use of vague, ambiguous, unstructured stimulus objects or situations in which the subject “projects” his or her personality, attitude, opinions, and self-concept to provide structure to a possible situation [25, 26]. This type of method has contributed significantly to many studies [27]. In a study on the ethnic identity preferences of Singaporeans, Chang [28, 29] designed a hypothetical reincarnation opportunity and asked the participants which race they wished to be born into. Chang’s findings suggested that making decisions relative to significant life events may provide important information about individual preferences. In the same vein, we inferred that selecting to reincarnate as a local/non-local inhabitant, choosing to marry a local/non-local projected future spouse, the willingness to pass on a dialect to an offspring, and emotionally reacting to discriminatory words against local people would detect whether inhabitants endorsed their own place of settlement without any biases (or at least free from social desirability bias).

Our hypothesis was based on Aesop’s idea from the story of the town and country mice and on the above-mentioned literature. Specifically, we hypothesized that subjective indicators, which measure how people assess their places of settlement, will decrease monotonically with the degree of urbanization, indicating a decrease in positive assessment with increasing urbanization.

The goal of our research was to increase our understanding of the impact of urbanization in China. We conducted two surveys to test our hypothesis. In the first survey, we developed direct and indirect subjective indicators to measure the assessments of inhabitants with regard to the effect of urbanization on their local place of settlement. The second survey further explored possible explanations for the findings that we obtained from the first survey.

## Study 1: Direct and Indirect Subjective Assessment

### Participants and Procedure

We used an anonymous survey method to encourage the participants to express their honest opinions and to not have to think about how to respond in a socially desirable manner. Instead of requiring the participants to sign a form, which would have contradicted the purpose of the anonymous survey, we allowed our participants to provide their verbal informed consent prior to participating in the survey.

The participants were allowed to opt out of the survey. This study did not elicit adverse physiological and psychological reactions from the participants but only measured their simple behavior responses. Therefore, the Institutional Review Board of the Institute of Psychology of the Chinese Academy of Sciences waived the need for written informed consent from the participants and approved our study. A nationwide, in-home survey was conducted from August to September 2007 on a stratified random sample of 3,716 Chinese inhabitants across the three tiers of settlements to measure their projected endorsements and perceived social ambiance of their settlements. The survey was conducted by a polling company. A stratified multi-stage random sampling technique was used to represent the three-tiered settlement hierarchy. The first stage of the random sampling occurred on the national level based on the GDP per capita, population size, and geographical distribution of each region (eastern, central, and western zones). Seven cities, seven towns, and ten country areas were selected as representative examples for the nationwide survey. The second stage randomly selected a cluster of addresses from each primary sampling area. Approximately 10 to 15 neighborhoods were randomly selected from each region as the sampling points, and approximately 10 individuals from each point engaged in extensive face-to-face interviews with a trained interviewer in the comfort of their homes for more than an hour. These interviews covered several issues, including demographic items and other items that were unrelated to the mental health of the participants (General Health Questionnaire [30]).

### Measures

#### Rated social ambiance

Six items (1A in S1 File) were developed based on the six aspects for which we rated the social ambiance [2, 21–23, 31]. For example, “The unfairness in society has been reduced” was used to measure fairness; “The public security is fine” was used to measure safety.

#### Projected endorsement

Four items were constructed to measure the projected endorsement of their areas by the participants. These involved the selection or deselection to be reincarnated as a local/non-local inhabitant, the choice to marry a local/non-local projected future spouse, the willingness to pass on their own dialect to an offspring, and their emotional reaction to discriminatory words. We hypothesized that the respondents who were more apt to endorse their current places of settlement would be more willing to be reborn as local inhabitants, to marry local spouses, to teach their offspring to speak the local dialect, or to become upset after hearing abusive or insulting words directed at the local people. Therefore, we developed a measure of projected endorsement based on these four issues (1B in S1 File).

### Results

**Fig 1:**
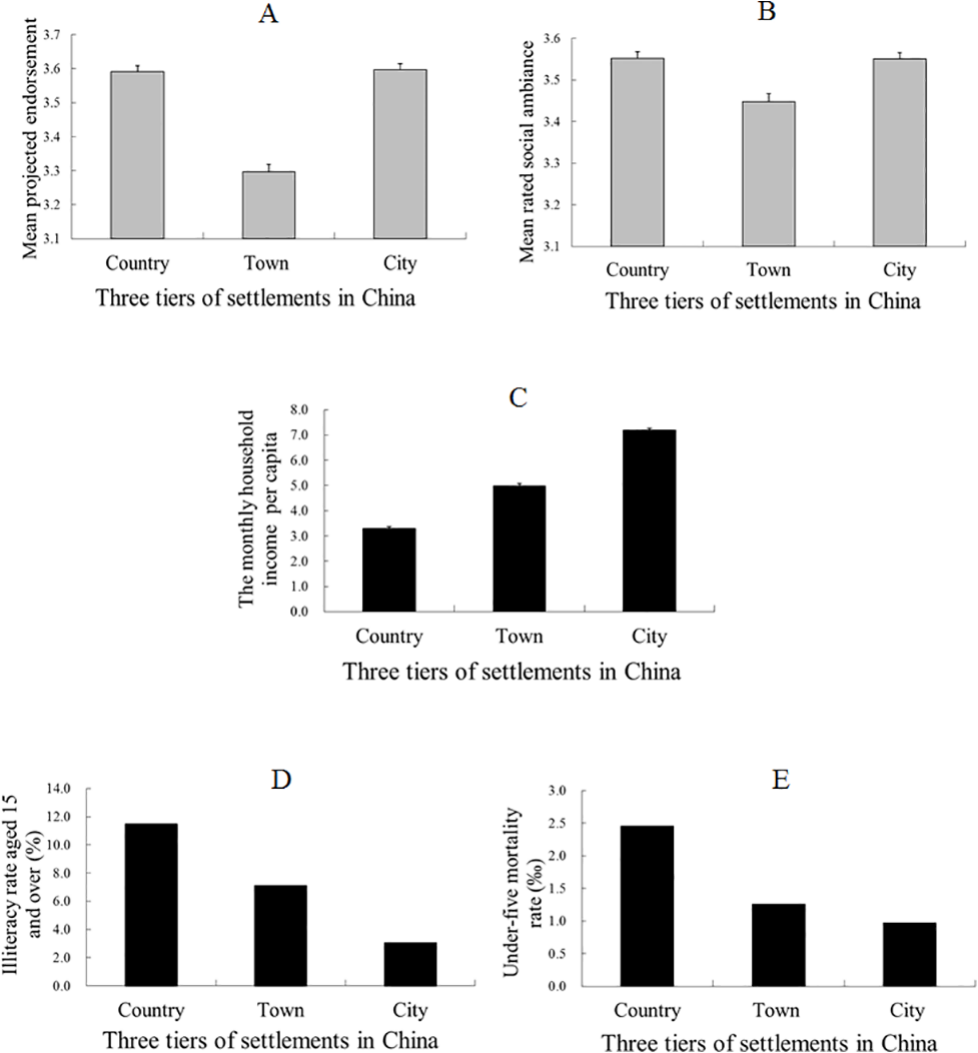
Data from the assessment of the effect of urbanization in Study 1. Panels A and B indicate the mean scores of the inhabitants’ projected endorsements and their ratings of social ambiance across the three tiers of settlements. A higher score indicates a more positive assessment of the effect of urbanization. Panels C, D and E indicate “the monthly household income per capita” (on a 13-interval scale, ranging from “under ¥500” to “¥5500+”), “the illiteracy rate for those aged 15 and over” and “the under-five mortality rate” (2006.11.1–2007.10.31, per 1000 births) across the three tiers of settlements. Bar heights indicate mean values. Error bars indicate standard errors. *Note*: The reported monthly household income per capita in Panel C improved significantly with the urbanization level in China (*F* (2, 3408) = 614.93, *MSE* = 7.83, *p* < 0.001, and η^2^ = 0.27, by ANCOVA). A post hoc analysis revealed significant differences between each settlement tier (*ps* < 0.05).

Table 1 shows the demographic data of the respondents. The scores of the two indicators (projected endorsement and rated social ambiance) were derived by averaging their responses across the corresponding four and six items, respectively. We tested one-factor and two-factor models to determine which of these models provided the best fit for the data [32]. The hypothesized two-factor model (GFI = 0.95, AGFI = 0.93, RMSEA = 0.08) yielded a better fit than the one-factor model (GFI = 0.90, AGFI = 0.84, RMSEA = 0.12), with a change in the chi-square of 900.77 (Δ *df* = 1, *p* < 0.001). These results indicated that the scales of the projected endorsement and rated social ambiance had adequate discriminatory validity for the hypotheses testing. The internal consistency of the projected endorsement and the rated social ambiance measured by Cronbach's alpha was 0.52 and 0.69, respectively, indicating that the questions of the scale converged to the same construct. The internal consistency of the projected endorsement was modest (Cronbach's alpha > 0.50) but met the suggested criterion, that is, a Cronbach's alpha >0.50 [33, 34]. Fig 1 summarizes the results of Study 1. To compare the soft (subjective) index with hard (objective) indices, we selected three hard indices based on the United Nations Development Program [35]. UNDP proposed three essentials to measure the levels of development, that is, whether people can lead a long and healthy life, can acquire knowledge, and can have access to resources for a decent standard of living [35]. Thus, we selected “the under-five mortality rate” which is commonly viewed as an important indicator of average population health [36] to measure whether people can lead a long and healthy life; selected “the adult illiteracy rate” to measure whether people can acquire knowledge; and selected “the monthly household income per capita” to measure whether people can have access to resources for a decent standard of living. The data for the under-five mortality rate and the adult illiteracy rate was obtained from the *China Population and Employment Statistic Yearbook 2008* [37]. The data for the monthly household income was, however, derived from the participants’ self-reports, due to a lack of monthly household income data in the *China Population and Employment Statistic Yearbook 2008* [37]. These three hard indices for the three tiers of settlements are shown in Panels C, D and E in Fig 1. Consistent with common perceptions about what life is like in various areas, certain socioeconomic status and health conditions, such as the monthly household income per capita, the under-five mortality rate and the illiteracy rate of people aged 15 and over (shown in Panels C, D and E of Fig 1), improved *monotonically* along with urbanization in China. However, the subjective assessments by the participants, which were measured through direct and indirect questions, did not follow the trend of the objective indicators. After statistically controlling for gender, age, and education, the rated social ambiance (direct measures) significantly differed between the participants from the three tiers of settlements (*F* (2, 3655) = 10.22, *MSE* = 0.34, *p* < 0.001, and η^2^ = 0.01, by ANCOVA). Fisher's least significant difference (LSD) post hoc test [38] revealed that the town residents rated their social ambiance lower than the ratings given by either country or city residents (for both contrasts, *p*s < 0.001). No significant difference was observed between the rated social ambiance of the two latter groups (*p* = 0.42). The projected endorsements (indirect measures) likewise significantly differed between the inhabitants of the three tiers of settlements [*F* (2, 3609) = 62.65, *MSE* = 0.44, *p* < 0.001, and η^2^ = 0.03, by ANCOVA] after controlling for gender, age, and education. An LSD post-hoc test revealed that the town residents gave a lower score for their projected endorsement than those given by the country and city residents (for both contrasts, *ps* < 0.001). No significant difference was observed between the projected endorsements of the two latter groups (*p* = 0.76).

**Table 1:** Demographic data in the two rounds of surveys.

			Percentage (%)		
			City	Town	Country
**Round I**			**(n = 1487)**	**(n = 916)**	**(n = 1313)**
**(n = 3716)**	**Gender**	Male	48.8	46.8	46.8
		Female	51.2	53.2	53.2
	**Age**	Under 19	2.4	5.1	5.3
		20–29	21.8	24.9	18.7
		30–39	25.7	25.3	23.9
		40–49	27.8	22.8	25.3
		50–59	20.8	19.5	23.5
		Over 60	1.3	1.9	2.2
		Unknown	0.3	0.4	1.1
	**Education**	Below senior high school	25.7	54.3	75.6
		Senior high school and above	74.2	45.0	22.5
		Unknown	0.1	0.8	1.9
**Round II**			**(n = 639)**	**(n = 399)**	**(n = 414)**
**(n = 1452)**	**Gender**	Male	44.9	47.1	49.5
		Female	55.1	52.9	50.5
	**Age**	Under 19	3.1	10.0	1.7
		20–29	21.4	34.8	15.5
		30–39	28.3	26.8	28.3
		40–49	24.6	16.0	26.1
		50–59	20.7	11.3	26.3
		Over 60	1.9	1.0	2.2
	**Education**	Below senior high school	32.1	34.1	88.6
		Senior high school and above	67.9	65.9	11.4

These results indicated that the city inhabitants did not rate their place of settlement and their rated social ambiance as either higher or lower than the country residents. Somewhat counterintuitively, the projected endorsement and the rated social ambiance of the inhabitants in towns, which were assumed to lie between the two extremes (country areas and cities) based on an assumption of finding a monotonical increase, were the most negative. Logically speaking, the assessment scores from people living in towns would be expected to be intermediate to those of the rural and urban residents, but it turned out to be out of order in this presumed sequence. Because of the illogical nature of these scores, we dubbed this phenomenon the “town dislocation effect” in the assessment of Chinese urbanization.

## Study 2: Possible Mechanism of the Town Dislocation Effect

Two possible explanations were proposed for the out-of-pattern score (OOPS) of the town settlement. First, the social support in country areas tends to be based on blood relationships, whereas the social support in cities tends to be based on professional relationships [39]. The basis of social support appears to change from blood relationships to professional relationships during the urbanization process [40–42]. The social support in towns could be lower compared to that of either the country areas or the cities because the blood relationships had been destroyed but the professional relationships had not yet been established in towns [39, 41].

Second, the residents of (developing) towns may face more uncertain situations than the residents of (undeveloped) country areas and/or (developed) cities. The development of towns and villages under the Small City Strategy is an important and unique feature of Chinese urbanization [43], which will potentially result in significant changes and unforeseen risks to the local residents. The reluctance of town residents to accept these proposed changes and their aversion to adventure have been assumed to affect their assessment of their places of settlement. Therefore, we hypothesized that the town dislocation effect may be mediated by social support, receptiveness to innovation, and a sense of adventure.

### Participants and Procedure

A second-round survey was conducted from October 2007 to November 2007 on a stratified random sample of 1,452 residents from the three tiers to test this second hypothesis. Using the selection procedure from the first-round survey, a different set of three cities, three towns, and three country areas was selected for Study 2.

### Measures

The rated social ambiance and projected endorsements of the participants were also collected in Study 2 (Cronbach's alpha for the rated social ambiance was 0.74; Cronbach's alpha for the projected endorsement was 0.58). Three potential mediators were measured in the second-round survey in addition to the indicators that were used in the first-round survey. Five items of social support were borrowed and modified from the perceived social support scale of Blumenthal et al. [44] and from the trust in people scale of Survey Research Center [45]. The respondents were asked if their friends or other people would provide them with advice, suggestions, and/or assistance if they were ever in trouble (2A in S2 File). The respondents answered these questions on a five-point Likert scale, with 1 denoting “definitely disagree” and 5 denoting “definitely agree.” The consumption of genetically modified (GM) food can be considered to be a high-risk behavior and has become a significant concern in China [46, 47]. Therefore, we assessed the attitudes of our respondents toward GM food to measure their receptiveness to innovation. The questions for measuring such attitudes were borrowed and modified from the study by Gaskell, Brauer, Durant, and Allum [48]. The respondents were asked whether they believed that genetically modified foods were useful for society, risky for society (the scale was inverted), morally acceptable, and should be encouraged [48, 49] (2B in S2 File). The respondents answered these questions on a five-point Likert scale, with 1 denoting “definitely disagree” and 5 denoting “definitely agree.” Another candidate variable considered for the second-round survey was loss aversion, which is generally considered to be the strongest component of risk aversion [50–52]. We obtained the measures for loss aversion by asking questions about hypothetical payoffs [53]. The respondents were asked to indicate the maximum (minimum) amount of money for a coin tossing game in which they would win ¥100 (¥X) if the coin landed on heads but would lose ¥X (¥100) if the coin landed on tails. The loss aversion coefficient was calculated by the ratio of sensitivity to losses versus gains. A higher loss aversion coefficient would indicate a higher level of risk aversion, of which loss aversion is the primary constituent (2C in S2 File).

### Results

**Fig 2:**
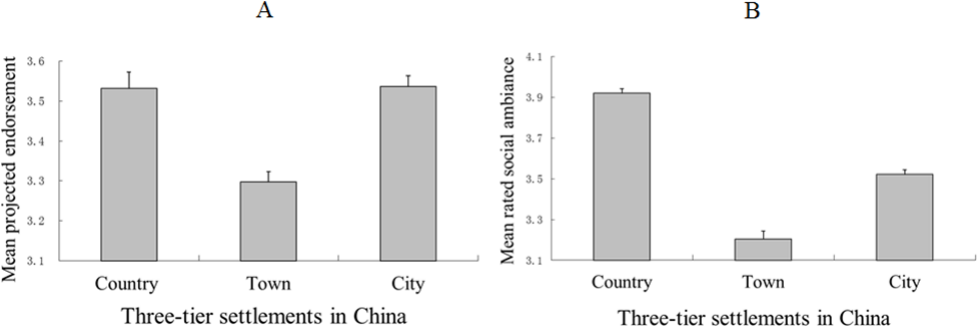
Data from the assessment of the effect of urbanization in Study 2. Panels A and B indicate the mean scores of the inhabitants’ projected endorsements and their ratings of social ambiance across the three tiers of settlements. A higher score indicates a more positive assessment of the effect of urbanization. Bar heights indicate mean values. Error bars indicate standard error.

A V-shaped pattern was observed in Study 2 (Fig 2) as it had been in Study 1. This result indicated the robustness of the town dislocation effect in Chinese urbanization. The respondents from the three tiers of settlements differed significantly in their assessments [*F* (2, 1446) = 15.00, *MSE* = 0.47, *p* < 0.001, and η^2^ = 0.02 for the degree of projected endorsement; *F* (2, 1446) = 106.47, *MSE* = 0.38, *p* < 0.001, and η^2^ = 0.13 for the rated social ambiance, by ANCOVA] after controlling for gender, age, and education. The respondents from the towns assigned lower scores to both the direct and indirect measures (means of 3.30 and 3.21 for projected endorsements and rated social ambiance, respectively) compared to the scores assigned by the respondents from the country areas and the city (means of 3.53 and 3.54 for projected endorsements, respectively, and means of 3.92 and 3.52 for rated social ambiance, respectively; *p*s < 0.001).

The scores for social support and receptivity to GM food were derived by averaging their responses across the corresponding five items (Cronbach’s Alpha = 0.74) and four items (Cronbach’s Alpha = 0.72), respectively. We tested one-factor and two-factor models to determine the discriminatory validity of these two variables. The hypothesized two-factor model (GFI = 0.98, AGFI = 0.97, RMSEA = 0.05) yielded a better fit than the one-factor model (GFI = 0.76, AGFI = 0.61, RMSEA = 0.20), which supported the adequate discriminatory validity of the scale.

Further analyses of the newly added measures revealed that the mean social support [*F* (2, 1446) = 27.72, *MSE* = 0.30, and *p* < 0.001, η^2^ = 0.11, by ANCOVA], receptivity toward GM food [*F* (2, 1273) = 11.61, *MSE* = 0.59, and *p* < 0.05, η^2^ = 0.02, by ANCOVA], and loss aversion coefficients [*F* (2, 1202) = 17.37, *MSE* = 12.80, *p* < 0.001, and η^2^ = 0.03, by ANCOVA] of the respondents from the three tiers of settlements differed significantly after controlling for gender, age, and education. An LSD post-hoc test revealed that the town residents assigned lower scores to social support than did either the rural or city residents (for both contrasts, *ps* < 0.05). Moreover, the rural residents assigned significantly higher scores to social support than the city residents (*p* = 0.04). LSD post-hoc test likewise revealed that the town residents had more receptivity toward GM food and exhibited less loss aversion than the city or country residents (for both contrasts, *ps* < 0.001). In addition, no significant difference emerged between the two latter groups in terms of their receptivity toward GM food (*p* = 0.06) and loss aversion (*p* = 0.12).

**Fig 3:**
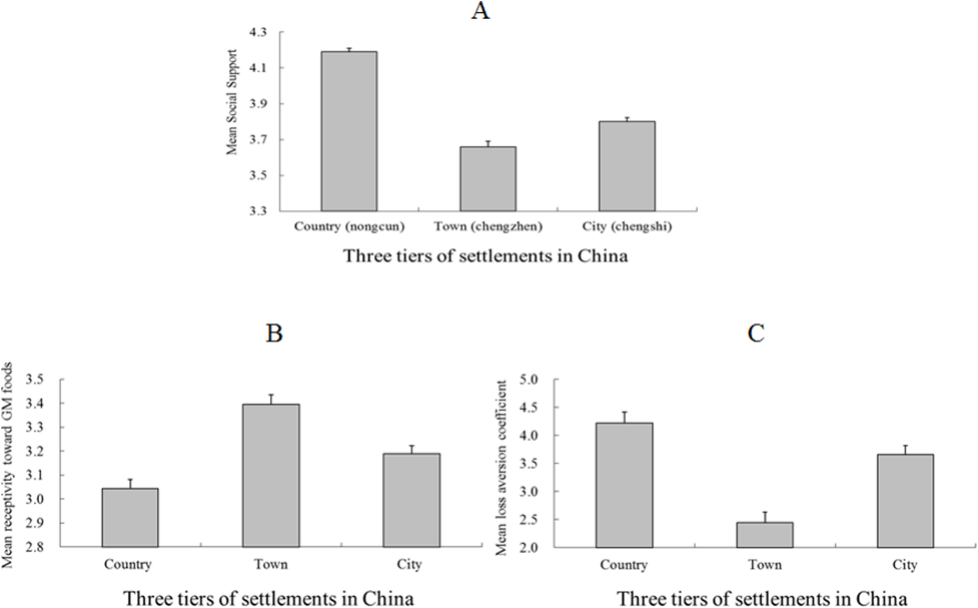
Data from the assessment of the effect of urbanization in Study 2. Panels A, B and C indicate the mean scores for social support, receptivity toward GM foods, and the loss aversion coefficient across the three settlement tiers. A higher score in Panel A indicates a more positive level of social support. A higher score in Panel B indicates a more positive receptivity toward GM foods. A lower score in Panel C indicates less sensitivity to the possibility of losing money than to the possibility of winning money. Bar heights indicate mean values. Error bars indicate standard error.

We observed a V-shaped town dislocation effect, which indicated that the town residents reported the lowest level of social support (Fig 3). However, we observed two unexpected reverse V-shaped town dislocation effects (Fig 3), which indicated that the town residents were very willing to embrace adventure and innovation. These results become logical if the town residents are viewed as rural–urban migrants, since the high aspirations of migrants are associated with their lower level of happiness [54].

We tested the mediation effect of social support, receptivity toward GM food, and loss aversion using the bootstrap method recommended by Preacher and Hayes [55] with gender, age, and education as control variables to explain the town dislocation effect on the assessment of urbanization in China. 95% CIs (confidence intervals) were obtained for all tests in the mediation analyses using a bias-corrected method with 5,000 bootstrap samples. This mediation effect for the three tiers of settlements on the rated social ambiance through social support was significant at the 95% confidence level (CI ranging from 0.040 to 0.078), and the mediation effect for the three tiers of settlements on projected endorsement through social support was also significant at the 95% confidence level (CI ranging from 0.027 to 0.058). The two other selected variables failed to provide a satisfactory explanation for the town dislocation effect, as shown by the fact that their 95% confidence intervals included zero. Specifically, the mediation effects between the three tiers of settlements and social ambiance were not significant with a 95% CI for receptivity toward GM food (CI ranging from -0.005 to 0.002) and for loss aversion (CI ranging from -0.003 to 0.007). Similarly, the mediation effects between the three tiers and projected endorsement were also not significant with a 95% CI for receptivity toward GM food (CI ranging from -0.0001 to 0.011) and for loss aversion (with a CI ranging from -0.002 to 0.002). Note that because the confidence interval did not include zero, we can conclude that the effects of the three tiers of settlements on the soft indicators (rated social ambiance and projected endorsement) were mediated by social support. This finding was supported by other studies that showed that social relationships [56, 57], social bonds [58], and community ties [59] were positively related to place attachment, which refers to the bond between individuals and their environments [60].

## General Discussion

This study developed and employed soft indicators to assess the effect of urbanization. We found that the soft and hard indicators measured different effects.

Because urban residents enjoy better health [61, 62], educational resources [62], and living standards [62] than rural residents, they are assumed to be happier to live in and more apt to endorse their local place of settlement. However, the two studies reported here tell a different story in that urban inhabitants did not assign higher ratings to soft indicators than those assigned by their rural counterparts, a finding which contradicts both common sense and our hypotheses. A similar discrepancy between soft and hard indicators was reported by Kahneman and Krueger [63], who found that the life satisfaction or happiness of people in China did not improve from 1994 to 2005 despite a 250% increase in their average real income. Knight and Gunatilaka [54, 64] found and analyzed a rural–urban divide paradox in China, which claimed that although the urban households were richer than their rural counterparts, the average happiness of the former was lower than the latter.

One of the types of soft indexes that were used in this study was a projective measure similar to those that have been used in numerous fields. Examples include the shopping list in consumer research [26, 65] and the affect misattribution procedure in attitude research [66]. Projective measures enter the private worlds of the participants to uncover their inner perspectives [26] and can measure their dependent variables [67, 68]. Given that the projective ratings that we developed are more likely to be free of social desirability bias than the direct measures that are typically used, it is relatively safe to believe that the town dislocation effect is real. After assessing the entire spectrum of the places of settlement from a subjective perspective, we found a town dislocation effect that those who had used objective criteria were likely to have overlooked.

The mediation analysis showed that the town dislocation effect could be accounted for by social support, a finding which was consistent with those of previous researchers. The presence of social relationships [56, 57], community ties [59], and close friends in the vicinity [69] can directly affect place attachment and place identity. People who could obtain more social support tend to speak more highly of their residence. Our research suggests that improving hard indices (e.g., socioeconomic status measures and health) was insufficient to increase people’s assessment of urbanization. Additionally, focusing on the social support network in towns could be important for facilitating the transition and improving people’s assessment at all stages of urbanization.

We used a bootstrap method to test the mediation effect between the three tiers of settlements and the soft indicators to explain the OOPS for the town settlement. This method boasts two advantages which improve its reliability in testing such mediation effects. First, the bootstrap method is more powerful and produces fewer Type I errors than other methods [70–73]. Second, unlike other methods of mediation analysis, generating a bootstrap CI does not require a standard error for any of the paths [74, 75]. The reliability of this method helped enhance the credibility of our results.

### The Future: Where Do We Go from Here?

China is on a path of inevitable urbanization. The urban population of China increased from 17.9% in 1978 to 44.94% in 2007 [37] and is expected to increase to 70% by 2050 [2]. Therefore, the welfare of the inhabitants must be improved throughout the urbanization process. The current study can increase the understanding of policy makers about the effect of their decisions on people’s lives. We suggest that urban development policies must balance economic growth against respect for beliefs about the factors that constitute the ideal living conditions for people. Inattention to soft indicators when assessing the urbanization process could result in misleading analyses and would be likely to fail to detect the town dislocation effect. Unfortunately, subjective indicators are seldom used in social policy studies because of their instability and incomparability. Because of these negative factors associated with subjective indicators, the skepticism of policy makers increases from the use of objective assessments to test objective substance (the least criticized) to the use of subjective assessments to test subjective substance (the most criticized) [17]. Getting participants to examine projected endorsements is used in an attempt to improve the credibility of “measures subjective substance using subjective appraisal.” Projected measurements, which are free of social desirability bias, can be expected to decrease the skepticism of policy makers and to benefit future investigations and assessments of the urbanization process.

The observed town dislocation effect also suggests that special attention must be given to towns and their residents. Towns have been considered the “workhorses” of Chinese urbanization for the past three decades [76]. Town residents reported the poorest assessment of their places of settlement. Thus, policy makers must integrate the public voice of these people into the formative, decision-making stages and consider adopting meaningful measures for the urbanization progress, which can effectively represent the real wealth of the life experiences of these people. The willingness of town residents to embrace adventure and innovation should prompt policy makers and planners to consider conducting public policy experiments in towns rather than in cities or country areas. Although this study is limited by its use of only one measure for each indicator, we believe that our findings will contribute to the improvement of the urbanization process in China.

## Supporting Information

S1 File 1A, Questions for Rating Social Ambiance. 1B, Questions for the Projected Endorsement.
(DOC)

S2 File 2A, Questions for Social Support. 2B, Questions for Receptivity toward GM Foods. 2C, Questions for the Loss Aversion Coefficient.
(DOC)
