# Supplementary material for: Town Mouse or Country Mouse: Identifying a Town Dislocation Effect in Chinese Urbanization
Source: PLoS One. 2015 May 14;10(5):e0125821. doi: 10.1371/journal.pone.0125821 (PMC4431860; doi:10.1371/journal.pone.0125821)
Supplement: S1 File — 1B, Questions for the Projected Endorsement. (DOC) [file pone.0125821.s001.doc]

**S1 Study 1**

**S1A Questions for Rating Social Ambiance**

| 1. This society will be increasingly just/impartial | | | | |
| --- | --- | --- | --- | --- |
| strongly agree | agree | indifferent | disagree | strongly disagree |

| 2. This society will be increasingly orderly | | | | |
| --- | --- | --- | --- | --- |
| strongly agree | agree | indifferent | disagree | strongly disagree |

| 3. People's social status will be increasingly equal | | | | |
| --- | --- | --- | --- | --- |
| strongly agree | agree | indifferent | disagree | strongly disagree |

| 4. The unfairness in society has been reduced | | | | |
| --- | --- | --- | --- | --- |
| strongly agree | agree | indifferent | disagree | strongly disagree |

| 5. This society is more tolerant than before | | | | |
| --- | --- | --- | --- | --- |
| strongly agree | agree | indifferent | disagree | strongly disagree |

| 6. The public security is fine | | | | |
| --- | --- | --- | --- | --- |
| strongly agree | agree | indifferent | disagree | strongly disagree |

**S1B Questions for the Projected Endorsement**

1. If you were to select or re-select a spouse, you would be

| very willing to marry a non-local spouse | 1 |
| --- | --- |
| willing to marry a non-local spouse | 2 |
| indifferent | 3 |
| willing to marry a local spouse | 4 |
| very willing to marry a local spouse | 5 |

2. If you have or will have offspring, you would be

| very unwilling to teach your offspring to speak the dialect | 1 |
| --- | --- |
| unwilling to teach your offspring to speak the dialect | 2 |
| indifferent | 3 |
| willing to teach your offspring to speak the dialect | 4 |
| very willing to teach your offspring to speak the dialect | 5 |

3. If you had a “second life”, you would be

| very willing to be reborn as a non-local inhabitant | 1 |
| --- | --- |
| willing to be reborn as a non-local inhabitant | 2 |
| indifferent | 3 |
| willing to be reborn as a local inhabitant | 4 |
| very willing to be reborn as a local inhabitant | 5 |

4. If an outlander used abusive or insulting words about the local people, you would be

| very happy | 1 |
| --- | --- |
| happy | 2 |
| indifferent | 3 |
| upset | 4 |
| very upset | 5 |
