# Supplementary material for: Town Mouse or Country Mouse: Identifying a Town Dislocation Effect in Chinese Urbanization
Source: PLoS One. 2015 May 14;10(5):e0125821. doi: 10.1371/journal.pone.0125821 (PMC4431860; doi:10.1371/journal.pone.0125821)
Supplement: S2 File — 2B, Questions for Receptivity toward GM Foods. 2C, Questions for the Loss Aversion Coefficient. (DOC) [file pone.0125821.s002.doc]

**S2 Study 2**

**S2A Questions for Social Support**

| 1. The people around me can help each other. | | | | |
| --- | --- | --- | --- | --- |
| strongly agree | agree | indifferent | disagree | strongly disagree |

| 2. I can find someone to talk to when I am in trouble. | | | | |
| --- | --- | --- | --- | --- |
| strongly agree | agree | indifferent | disagree | strongly disagree |

| 3. My friends can give me some advice and suggestions whenever I am in trouble. | | | | |
| --- | --- | --- | --- | --- |
| strongly agree | agree | indifferent | disagree | strongly disagree |

| 4. I have some friends on whom I can rely whenever I am in trouble. | | | | |
| --- | --- | --- | --- | --- |
| strongly agree | agree | indifferent | disagree | strongly disagree |

| 5. Most people will do everything in their power to help others who are in trouble. | | | | |
| --- | --- | --- | --- | --- |
| strongly agree | agree | indifferent | disagree | strongly disagree |

**S2B Questions for Receptivity toward GM Foods**

| 1. To what extent do you agree or disagree that genetically modified foods are useful for society? | | | | |
| --- | --- | --- | --- | --- |
| strongly agree | agree | indifferent | disagree | strongly disagree |

| 2. To what extent do you agree or disagree that genetically modified foods are risky for society? | | | | |
| --- | --- | --- | --- | --- |
| strongly agree | agree | indifferent | disagree | strongly disagree |

| 3. To what extent do you agree or disagree that genetically modified foods are morally acceptable? | | | | |
| --- | --- | --- | --- | --- |
| strongly agree | agree | indifferent | disagree | strongly disagree |

| 4. To what extent do you agree or disagree that genetically modified foods should be supported? | | | | |
| --- | --- | --- | --- | --- |
| strongly agree | agree | indifferent | disagree | strongly disagree |

**S2C Questions for the Loss Aversion Coefficient**

Version 1: Imagine you have been asked to play a coin tossing game in which you will win ￥100 if the coin lands heads, and you will lose ￥X if it lands tails. Please indicate the maximum amount of X that you are willing to accept to play this game.

Version 2: Imagine you have been asked to play a coin tossing game in which you will win ￥X if the coin lands heads, and you will lose￥100 if it lands tails. Please indicate the minimum amount of X that you are willing to accept to play this game.
